# Life in Hospital

**Published:** 1887-06-04

**Authors:** 


### Life in Hospital.

By our Special Commissioner.

### Mrs. Johnson Argues the Point.

"The best thing you can do, Mrs. Johnson, is to go into the hospital," said the doctor.

Mrs. Johnson was the wife of a carpenter, a middle-aged woman with four children to look after; and the idea of leaving them—not to speak of Tom, her husband—to the chances of every accident that can befall a family when the wife and mother is absent, was very terrible. But for more than a year her health had been giving way. She felt her daily work to be a labour beyond her strength, and she could not help getting terrible attacks of low spirits, which puzzled the children greatly, and distressed Tom not a little. He tried to cheer her, but it was of no use; she only grew more depressed and tearful, and sometimes she was cross as well. When she at last consulted a doctor, he told her that she was suffering from a tumour, which should be removed as soon as possible, or her life must pay the penalty; and he advised her to go to the hospital.

"It is usually necessary to get a letter of recommendation from a subscriber to the funds of the hospital," he said; "but the governor never refuses to take in a case of real importance, and will certainly make room for you if I ask him."

"Thank you," said Mrs. Johnson, rather sharply; "but I'd rather not go to any hospitals. I've never took anything in charity yet, and I don't want to begin now."

### A Good Investment.

"I am very sorry that the hospital should be altogether a charity to you," answered the doctor; "but it's your own fault. If you had subscribed something to its funds every year since you were married, you wouldn't think that you were taking charity, but benefiting by a sensible investment."

"Working people haven't got money to spend on subscribing to things like that," she answered. "With four children to bring up and educate decently, and provisions the price they are, and the societies to pay into, we've got enough to do just to keep going."

"What societies are you speaking of?" asked the doctor.

"The Foresters, that would give Tom ten shillings a week if he was laid up and not able to work; and the Burial Society, that gives you enough money to pay for a decent funeral if any of your family dies."

"Now, Mrs. Johnson, isn't it strange that you, who are a sensible woman, should have been giving money for years to a society that will profit you only when those you love die, and yet have never thought of subscribing to an institution that is meant to bring them back to health, and may postpone your needing that burial money for many a year? The hospital is in one way very like your societies. It will take you in when you are ill, or have met with an accident—its doors are open day and night to the sick and hurt—and give you the best doctoring, the best nursing, and the best care of every kind that you could possibly get. But it is much more generous than they are. Those who manage it don't say that you shall not profit by it because you haven't paid into its funds. They take in the most destitute, and give them every opportunity of regaining health that science and kindness can invent; and don't even demand payment from people like you, who are able to keep your head above water, and put by a little for future emergencies. But your pride—and a very good, proper pride it is, if you take to heart the lesson it teaches—makes you resent the idea of taking all this in charity; you like to feel that you pay for what you get. You can do that only by giving something to the hospital funds every year. Just you save up as much, or even half as much, as you pay to the Burial Society, and put it into the offertory-bag on Hospital Sunday every year, and you'll have the satisfactory feeling of paying, to the best of your ability, for the benefits you will have got from it; and if either you or any of your family require to go to it again, you will feel that you are not taking charity, but getting something you have, in part at least, paid for."

### "Can't it be done at Home?"

"What you say is very true, no doubt, doctor; and somehow I had never thought of it in that light before. I always took the hospitals to be places which the rich folks kept up for the poor ones, and didn't think that it was ever really meant for them, like me and my Tom, to help in it and profit by them. But still I'd rather be in my own home, and be cured there."

"You're wrong in wishing that," said the doctor. "I doubt if it would be possible, even as a matter of expense, not to speak of anything else."

"I know that operations cost a good bit; but I daresay we could manage it," she answered.

"I doubt it. Remember that the removal of a tumour such as yours isn't a task that every man can attempt. It would be easy to count up the number of surgeons in England who venture on it; for it cannot be done without special skill and years of thought and study devoted to only one class of diseases. The doctor who does such operations at the hospital has given up almost all other work, and devotes his whole time and care to the relief of such cases as yours."

"Then, of course, he would require to be well paid," reflected Mrs. Johnson. "How much might his charge be?"

"If you were a rich lady in the West-end he would probably ask a hundred pounds."

"My!" exclaimed the poor woman, utterly taken aback at the mere mention of such a sum.

"And that isn't all," the doctor went on. "You would require two skilled nurses, thoroughly trained and experienced in such cases; for good nursing is half the battle. And you would need more quiet than you could get in your little four-roomed house, where you would always be hearing the children romping about, or little Tommy crying when his leg hurt him; and wouldn't be able to take the rest you need."

"That's true; but it's just the children, and especially my poor little lame Tommy, that I can't bear leaving. Tom's sister would come to take care of the house; but I know that little Tommy would cry for his mother."

"Better he should miss his mother for a few weeks than lose her altogether. You are a sensible woman, Mrs. Johnson, and know that the only cure for a case like yours is an operation, which, even if you could afford it, could hardly be safely performed in your own home; but which can be done in the hospital with every probability of success, and without costing you a penny. Don't hesitate; make up your mind to go."

### Students Round the Bed.

"But," she went on, "I have heard of people that went into the hospital, and were left to the care of students. You know, one doesn't like to be seen to by a young man who doesn't know his work; or to have a whole lot of them hanging round one's bed."

"You may be sure," the doctor answered, "that no patient is left to the care of a student alone, though one may be entrusted with the renewal of bandages and other dressings, and may be given the special charge of the case under the doctor's supervision. And you must remember that these young men are learning their profession. You would not have them sent to the world to attempt the cure of diseases without having seen these same ailments before, and learned the treatment of them. The hospital is the only place where they can get this knowledge; they study every form of disease under experienced doctors, and so are qualified when they go away to undertake the work of their profession with the probability of succeeding in it."

"That may be, sir; but I don't know that it is very pleasant for the patients."

"I assure you that many of them look forward to the visit of their student-dresser, and would feel quite depressed if they didn't see his bright face. And you must remember that this, being made a means of imparting knowledge, is the only price that most of the patients pay for all they get in the hospital; and that the students do a great deal towards keeping up the institution. Each of them pays a large sum for the right to study in the hospital; and these fees in some cases form no inconsiderable part of its income. Without that money its usefulness would be greatly lessened. And you may be sure that no hospital doctor ever permits, far less encourages, an unwarrantable examination of any patient. You may take my word for it that you will have no cause for complaint on that score in the hospital."

### Silenced—Not Quite Convinced.

Mrs. Johnson was not wholly convinced, but her case was so serious that she did not venture to refuse the only means of remedy open to her, and within a few days she found herself within the walls of the hospital. Dr. Fletcher having secured her admittance, she was able to go straight to the ward she was to occupy, without going to the patients' waiting-room, where cases that have not been recommended are first seen, and where slight ailments, both medical and surgical, are daily treated. Every morning a great number of both sexes may be seen trooping to the hospital, some with bandages on head or arm, others, whose ailments have not such obvious tokens, betraying their errand by the bottles they bring for their medicine, usually big black bottles in which even a pretty large supply of medicine seems lost. Thousands are thus attended annually, and in the hours set apart every day for seeing out-patients the doctor and his assistants have a busy time, which tests not only their skill, but their temper and patience, when some invalid not used to clear definition is trying to explain the symptoms he feels. Sometimes, if he is unduly prolix, he may be told good-humouredly to "hurry up"; for there are perhaps fifty other patients waiting their turn, some of them perhaps much more seriously ill than he; but everything he can tell that really affects his condition is listened to not merely with courtesy, but with interest.

There were five resident house-physicians and five house-surgeons attached to the institution where Mrs. Johnson was to be treated, in addition to the visiting physicians and surgeons, each of whom attended to the out-patients one day a week, besides paying a daily visit to the wards under their charge; and there was enough for all of them to do, for the hospital stood in a poor district, with works and factories of all sorts in its immediate vicinity; and as well as the numerous cases they had of diseases induced by insanitary conditions of life—bad air, ill-built houses, insufficient food—there were accidents without number continually coming to them, some of the most serious nature, some comparatively trifling, but all needing the immediate and careful attention they received. Only those which rendered the patient completely helpless could be treated entirely within the hospital, for the resources of the institution were limited. Large as it was, the five hundred beds it held formed a very limited accommodation for a district whose population was over 100,000, many of whom were, from the nature of their employment, peculiarly liable to accident or disease.

### The Pavilion Plan.

The hospital was built on the pavilion plan; that is, the eight blocks of building of which it was composed were united only by long corridors on the ground-floor, so that the risk of infection spreading from one ward to another was reduced to a minimum. Infectious cases were not, however, received in this building, but were taken to the Fever Hospital in connection with it, which was a considerable distance away from the hospital itself. Sometimes, however, patients suffering from a non-infectious disease when they were admitted, would develop symptoms of an infections one. In this case they were taken to the Observation House, a small building standing by itself within the hospital precincts, where they were kept till they were cured, or till all danger of infection had passed away.

The corridors were light, cheerful, and well-ventilated. They were kept warm by hot-air-pipes; and at regular distances there were to be seen hanging on the wall coils of indiarubber hose attached to brass taps in connection with the main water-pipe, so that in case of fire breaking out every appliance was at hand for extinguishing it. These coils of hose were also to be seen on the staircases, and in the short lobby at the end of each ward, off which opened the ward scullery, the doctor's sitting-room, the nurses' rooms, and two private wards.

### A Model Ward.

One great advantage of the hospital being built on the pavilion plan was that every ward occupied the whole width and depth of a block. Thus there were windows on each side of it, so that no patient could be unfavourably placed with regard to light. A thing which struck Mrs. Johnson the moment she entered the ward was that there was a window at the end of it also, just above the fireplace, which puzzled her a little. She wondered what had become of the chimney; and it was some time before she found out that the chimney went up within the wall at one side of this mantel-window. "It's the cleverest thing I ever saw," she said, "and looks every bit as well as the finest mirror a rich lady could put in her room."

But indeed the whole ward struck her as something much finer than she had ever seen—it was so very bright and cheerful, which was the last thing she expected a hospital to be. There were twenty-four beds in it, twelve on each side, standing between the windows. They had all bright scarlet quilts, which formed pleasant spots of colour against the light walls, on which were hung bright-coloured prints and one or two good engravings, and each was provided with the neatest table Mrs. Johnson had ever seen. It was of light polished wood—"and if ever I saw tables that were kept bright, it's these ones," she said to herself—and was narrow, not more than a foot and a half in width. But it was sufficiently long and high to go right across the bed, and ran so easily on its castors that the occupant of the bed could pull it towards or push it away from her with very little exertion. Down the middle of the ward ran long tables, on which stood ferns and flowering plants in pretty china pots, interspersed with glasses of cut-flowers. Two probationers in neat print dresses, with pretty caps and large aprons, were moving about the ward. They were young and pleasant-looking; and at the end of the room was another lady in a dark gown, with a cap slightly different in shape from that of the others, who had, the new patient thought, the sweetest face she had ever seen. This was Sister Grace, the sister in charge of the ward.

### Sister Grace.

"She isn't a bit what I expected a nurse to be," Mrs. Johnson said afterwards. "Somehow I had thought she would be old and ugly, and look as if she sometimes took a drop too much, like the old women in that story-book Tom bought." Indeed, many people who have not read *Martin Chuzzlewit*, as well as those who have, seem to think that Mrs. Gamp is a true and common type of nurse. If any creature so vulgar, ignorant, and selfish was ever entrusted with the care of the sick, she no longer fills such a position. She is an almost totally extinct species, and the last place where any trace of her is likely to be found is a hospital, where the nurses are frequently ladies both by birth and education, who have gone through careful training and severe discipline to fit them for a work which they pursue much more from love for and interest in suffering humanity than from any idea of personal gain. The hard work given to probationers, and the anxiety and thought that devolve on the staff-nurse, are almost certain to weed out all who do not possess a real innate capacity for the work they have chosen.

Sister Grace was almost everything that a nurse should be. Her step was noiseless and her voice sweet. She was never in a hurry and never out of temper, no matter how exacting and trying her patients might be; and she had always a pleasant word for each, encouraging them to patience, quiet endurance, and faith in God. She came forward now to meet Mrs. Johnson, who was leaning on the arm of Aunt Ellen, Tom's sister, just as if she were an old friend. "We have been expecting you," she said, with a smile. "This is your bed at the end of the ward, next to the fire, so I think you'll be comfortable. We are going to put you in the private ward in a day or two"—this was after the operation took place, but Sister Grace did not mention that—"but the doctor thought you had better come in here first." The doctor knew that just at first the patient was sure to be nervous and frightened, and wished her to get over her fear and become used to the situation before she underwent the shock of the operation.

Aunt Ellen went away, having received permission to come back on Sunday to see her sister, and to bring little Tommy with her. Then Sister Grace herself helped Mrs. Johnson to get to bed, and listened sympathetically and spoke cheerfully when the poor woman began to cry, and to predict that she would never see Tom and the children again. She stopped, however, in the midst of her grieving to exclaim about her bed. "Is it hair or feathers?" she asked. "For I never lay on anything so comfortable." "It is a water-bed," answered the Sister; "we find them better than anything else for patients who have to lie in bed for a long time."

### No Respect of Persons.

"But do you give these to poor people who have never been used to a soft bed all their lives?"

"Certainly, if they require it. The hospital does not make any distinctions between patients; all get just what they need; and often those who have been least used to comfort need it most. Quite a large number of our patients are poor maids-of-all-work, who are simply worn out with over-work, though some have injured themselves by pulling about furniture too heavy for them. The treatment they really require is simply rest, a good bed, and nourishing food. These soon make them all right. The girl in the bed opposite is one of those. She is getting well now, and is able to be up part of the day, and she is always so anxious to help us. She begs me to give her some dusting to do, or to let her clean some of the surgical instruments; and I really find it difficult to keep her from knocking herself up again in her longing to be useful."

### A Melancholy Night.

That first night in hospital passed rather drearily for the new patient. Her thoughts wandered back to those at home; and the vision of Tom sitting by the fire alone, while Aunt Ellen put the children to bed, and taught them to add to their prayers a petition that "dear mother might soon be well again," brought the tears to her eyes. Then the present scene was very strange to her. The long ward, with its double row of beds, shown faintly by the flickering fire-flame, the lights turned low, and the night-nurse flitting softly about, administering medicine or smoothing a pillow here and there, impressed her as something new and sad. The girl in the bed opposite, of whom Sister Grace had told her, was a very restless sleeper. Several times during the night she started up from her pillow, saying "she must get up at once, for this was washing-day, and her mistress would be very angry if she hadn't her work half done by breakfast time"; and the nurse had some difficulty in soothing her anxiety. Towards morning, too, the patient in the next bed to Mrs. Johnson woke up, moaning with pain. The night-nurse came to her, but saw immediately that there was something seriously wrong, and called Sister Grace and the house-surgeon. In a few minutes they were on the spot, and the doctor decided that a small operation was necessary at once. The poor woman uttered a suppressed cry when the night-nurse came to carry her into the doctor's room where the operation was to take place. "Don't touch me," she cried, "I'm in pain all over." But Sister Grace came near and said, "Let me carry you, Jessie; I won't hurt you," and lifting the patient up in her arms, carried her right out of the ward without causing her to utter a single moan.

"How does she do it?" asked Mrs. Johnson, for she saw that Sister Grace was small and slight, while the patient was a tall, big young woman, nearly twice the Sister's weight. "Oh!" answered the nurse, "she knows exactly how to take up a patient. Our Sister never hurts anybody."

In about an hour Jessie was brought back, not yet wholly recovered from the effects of chloroform. A probationer sat by the side of her bed to attend to her, and a screen was put round it, so that Mrs. Johnson could not see how she was getting on; but she heard her talking occasionally in a way that puzzled her a little, complaining that she could not find the chalk for her shoes, and asking if the rope was new and strong.

"Who is she?" asked our patient of Sister Grace, who came to inquire how she felt.

THE TIGHT-ROPE DANCER.

"Jessie? She is the daughter of a showman, and used to dance on the tight-rope," answered the Sister. "She fell from the rope once and hurt herself; gave no thought to the injury she had received, and so made herself very ill. She has had a very bad time since she came here, but she is wonderfully brave; looks cheerful at all times, and never cries out if the pain is at all endurable."

"Now! to think of my being not two yards away from a tight-rope dancer!" exclaimed Mrs. Johnson, who, as the wife of a respectable artisan, considered herself very much superior to anyone of that class. "You know, Sister Grace, ma'am, one doesn't just fancy people who do that sort of work; it doesn't seem quite respectable."

"If it isn't respectable to do it, one can't respect the people who go to see it done," answered Sister Grace. "And Jessie herself is a bright, happy, good girl, who is very fond of her father and wonderfully proud of him. When she is a little better she will tell you, I have no doubt, how well he plays the fiddle, and how much people think of his performances."

"And is she to go back to the rope-dancing when she is well again?"

"I don't think she will be able for it; but the difficulty is that she is fit for nothing else; she has had no training in any other work. But I will apply to the Association and try to get some other occupation for her."

A MUCH-NEEDED SOCIETY.

"What Association?"

"The Women's Christian Association. The members of it take an interest in young women, try to get situations for them, and to see that they are not led into temptation of any sort. As there are branches all over America and the Colonies, as well as in Britain, they can often help girls to emigrate who have no home or friends here. I frequently ask them to help me in disposing of what the doctor calls my visitors."

"Who are they, Sister?"

"Patients who are dismissed as cured, and have no home to go to. The doctor often allows me to keep them for a day or two, in order that I may see if I can't get a situation for them, and then the Association helps me greatly."

"But I should think some of them hadn't clothes good enough to go to a decent situation in."

"Then I have to apply to the Samaritan Society, who provide clothes—not always quite new ones, but very good—and often pay for a railway ticket if the patient's home is at a distance."

"And are these societies connected with the hospital?"

"Not exactly. None of the hospital funds are spent on things like that—unfortunately, there are none to spare for such objects; but they often hear in it of cases they can help, and we—the matron and the sisters—are only too glad to co-operate with them for such a purpose. I often wish more ladies knew of the Samaritan Society. If they sent some of their half-worn, old-fashioned clothes to it they might do a good work, and clear their wordrobes at the same time. That, however, is only a part of the work done by the Society. They give artificial limbs and other surgical appliances to poor people, help to support convalescent homes, and occasionally make small donations cf money to patients leaving the hospital. Often, you know, a very small sum, a few shillings, will keep a man till he gets work, and save him from starving, or going to the workhouse."

"That's a real useful Society."

SURGICAL APPLIANCES.

"It is; and there's plenty of need for it. It is said that one out of every ten persons in London requires to wear some artificial appliance. There are several operations, after which the patient must inevitably wear a truss or a belt, and there's no use our attempting them if we can't follow up the cure with the present of one of these appliances. There are two Surgical Societies, each of which distribute about 5,000 appliances every year—things of every kind, from artificial legs to spectacles."

"Spectacles! can't people buy them for themselves?"

"Not always. There are some diseases of the eye which require spectacles of special make, which can only be obtained from a good optician, and cost considerable sums. A pair of spectacles that don't exactly fit the case may strain the sight, and finally cause blindness. The Hospital Sunday Fund gave £1,600 to these societies last year; but, unfortunately, surgical appliances must be good and perfectly adapted to their purpose, and so are dear."

EARLY MORNING IN THE WARD.

The morning passed away quickly. The patients had breakfast served by the probationers—tea, cocoa, or milk, according to the needs of each and the orders of the doctor, and the ward was made ready for the visit of the house-surgeon. Mrs. Johnson considered herself a good house-wife, but she admitted that she had never seen work so neatly, thoroughly, and methodically done as it was here. The floor looked as if it might stand the old-fashioned test—"You might eat your dinner of it," as far as whiteness went, and not a speck of dust was allowed to linger anywhere. Nothing struck our observer so much as the dexterous way in which sheets were changed on several of the beds. There was no jolting, or lifting of the patient. Half the sheet was rolled up, and the rolled edge was placed in the middle of the bed close to the patient, who needed only to turn from one side to the other. This brought her to the smooth side of the sheet; then the rolled half was smoothed out, and the thing was done. "That's a thing worth knowing," said Mrs. Johnson to herself. "I remember when poor Tom broke his leg, how I used to hurt him when I put clean sheets on his bed; he'd never have felt it if I had known to do it in that way."

### The House-Surgeon.

Then the house-surgeon came in, and went round to each bed, accompanied by Sister Grace. At first Mrs. Johnson was afraid of his grave, keen face, but he spoke so kindly and cheerfully, and as if he knew her quite well, that she got over her fear and was able to answer his questions without hesitation. Then he went on to see Jessie, who saluted him with, "I'm awfully sorry, doctor, that I spoiled your night's rest as I did. I tried to keep quiet till morning, but the pain was too bad, and I had to cry out."

"I am glad you did, Jessie," he answered; "you needed the proverbial stitch in time, you see; and it might have been a serious business if it had been left undone for a few hours."

"Now, isn't that nice of him?" said Mrs. Johnson to herself. "And didn't one of the neighbours tell me that the hospital doctors would never do a thing for one out of their regular hours? But Mrs. Brown was always given to talking of things she knew nothing about. I'll let her know the truth when I get home again."

After the doctor's visit was past several of the patients who were nearly well got up and sat round the bright fire, reading or working. One of them came to Mrs. Johnson, who was lying watching with interested eyes. "Would you care to look at this book?" she said. "It's very nice; it is one of the Flower Mission books."

"I don't know that I care much about missionary books," answered Mrs. Johnson; "they're all about black men eating up poor helpless Christians, and the like."

"You don't understand what I mean. It isn't about that at all; it's a nice English story, and as interesting as the *Family Herald*, though it's just about working people. But what I meant was, that it is left by the Flower Mission ladies. They bring flowers in the summer—real wild-flowers often, and in the winter they bring books instead. But just you try the story; I know you'll like it." And so saying, she put *Silas Marner* in her hands.

### "To Pay for your Keep."

"I see some of you are sewing," Mrs. Johnson remarked; "I suppose you are set to do that when you are able to help pay for your keep, as they are in workhouses."

"No, we're not," answered the woman she spoke to, with a touch of indignation. "I've been here three weeks, and have never been asked to do a thing for anybody; but it's very dreary lying quite idle when you aren't feeling very weak, and I begged Sister Grace to give me something to do for her. I meant for herself, but she wouldn't let me do that; it was against the rules for the patients to work for the nurses, she said. However, she let me help in the ward, mending a little—darning sheets, putting on strings and buttons, and so on. Then a lady who visits the ward every week—she comes and sings to us, and says a word or two to each of us—asked me if I wouldn't like to knit myself a pair of stockings; and when I said I would, she got me wool, and showed me how to do it. Some of the patients get their friends to bring in the materials for their work; but all they do is of their own free will; nobody ever bids them do it."

The novelty of her surroundings made that day pass quickly, and Mrs. Johnson resolved to ask Aunt Ellen when she came the next day to get her some calico and sewing materials. It quite comforted her to think that she need not be entirely useless while she was in the hospital, but could make some things for the children, which she had not time to do at home. She did not foresee just then that it would be some time before she could do any stitching after the operation. She shrank a little when the visiting physician came in, followed by his students; but she was surprised to see how pleased all the patients were to see them, and how courteously the young men behaved. "Nothing's so bad as I thought," she said.

The next day was Sunday. A special quietness seemed to reign in the ward; only Jessie, who was better to-day, began to talk of the Sundays she had spent in the country in the family caravan.

"We had three performances on Saturdays generally, so it was a real treat to have a day with nothing to do, but just take a walk if you were so disposed, or lie in bed if you weren't. And we always had hot dinners on Sundays too; it was the only day mother had time and quiet to cook it. You can't manage a joint or a saucepan very well when the caravan is jolting over the road; and when we got to a village we had to set up the tent, and give a performance."

"And did you like that sort of life?" asked Sister Grace, who was near.

"Yes, in summer. In winter we mostly kept to the towns, so that we mightn't have to travel so often."

### Amateur Chaplains—Sunday Afternoon.

In the afternoon there was a new feature. Two of the students who had been in the ward on the previous day, came up and held a short service. One read some passages from the Bible, the other prayed, and the patients, such of them at least as were equal to such exertion, joined in singing two or three cheerful hymns. Before they concluded, Sister Grace went up and said a few words in a low voice, whereupon one of them turned towards Mrs. Johnson, and began to sing—she felt sure it was specially for her—

"Be still, my soul; the Lord is on thy side,
Bear patiently thy cross of grief and pain;
Leave to thy God to order and provide,
In every change He faithful will remain."

Tears were in the patient's eyes when he concluded, but they were tears of contentment and glad resignation to the divine will.

He came up and spoke to her after the service was over, and it seemed to her that never in her life had anyone said such kind and comforting words; she could hardly realise that they were uttered by a bright-faced young man, little more than a boy. She had somehow thought, as many people do, that religion was really valued only by women and old people, who had not much happiness left to expect in this world; she had not regarded it, as this young student seemed to do, as a thing to guide strength and manhood by. He was still by her bed when Aunt Ellen came with little Tommy, and with big Tom as well, the latter looking rather nervous and out of place while he tried elaborately to keep his boots from creaking as he walked up the ward; but quite determined to see if his Mary was comfortable, and to take her away at once if she wasn't.

"What's the matter with you, little man?" asked the student, as Tommy came limping up after his father. "Hip-joint disease, surely. You ought to come into the hospital, and we'd make you all right. Will you let us try to cure him, Mrs. Johnson?"

"I don't know," she answered dubiously; but Tom spoke out stoutly—

"If you make the missus better I might let you try it, but I'd like to be sure that she's right before you meddle with the child."

### No Cakes allowed.

After the student had left them Aunt Ellen opened a basket she had brought with her. "I made a cake for the children, Mary," she said. "Not a very fine one; but I thought they ought to have something nice for tea the first Sunday their mother was away from them. I brought a bit with me for you to taste, but I saw a notice as we came into the ward, that patients' friends must not bring anything in the way of food into the hospital."

"It's sheer tyranny," said Tom, who was a Radical, and did not believe in authority, though he liked to be master in his own house.

"I wonder what Sister Grace would say," remarked Mrs. Johnson, and, happily, at that moment, the Sister drew near, and the question was submitted to her.

"I think you may take it," was her verdict. "We are obliged to make the regulation you noticed, because people so often insist on bringing their friends the things that do most harm to them. More than once I have known a patient who was forbidden to have any heating food have a serious relapse through drinking spirits that had been smuggled in by a well-meaning but ill-advised friend. On the other hand, I know that things that have been brought from home often seem more appetising than those the hospital provides; and if they won't hurt the patient we always permit their acceptance. You have done quite right in asking my advice, and I am sure I can trust you never to bring anything to Mrs. Johnson without telling me. Only don't bring anything of the nature of alcohol; you may be quite sure that the hospital will provide her with as much as she needs."

"You keep the place very pretty, ma'am," remarked Tom, trying to overcome a certain awkwardness that was apt to overcome him in the presence of strangers. "It must cost a good bit to get all those plants in these pretty pots."

"It costs us nothing but the trouble of watering them. They are all gifts; some of them from patients and their friends, some from people outside. For the doctor of our ward is much attached to his hospital work, and his rich patients know that they cannot show their gratitude to him in a way that would please him better than a gift to the hospital. All our pretty things come in that way."

"I do a little in the gardening way myself in my spare time," said Tom, getting more nervous than ever. "If you wouldn't think it too small, I should be very pleased if you would accept one of my plants. I have got some fine chrysanthemums just beginning to flower."

"That *is* kind of you," said Sister Grace. "A gift of that kind is a real charity, and gladdens many more hearts than you know."

"No charity at all," answered Tom. "If you can make my missus well and strong again, it's no more than my duty to do all I can for the hospital, and I'll do it, you may be sure."

### The Operation.

Of the next day Mrs. Johnson could not recall much, for it was the day of the operation. She had no breakfast that morning, for it is not advisable to eat just before chloroform is administered; and immediately after the house-surgeon's visit she was taken into the small private ward at the end of the large one. It was next the house-surgeon's laboratory, which was furnished with shelves of medicine-bottles, and cases of lint and other dressings; and opposite Sister Grace's sitting-room, so that help was always near any patient in it. The Sister's bedroom, too, was in the same corridor, so that neither by day nor night was she far from the patients under her care. The private ward had double doors, the inner one covered with thick baize, so that no sound might enter from the corridor, as it is often on absolute quiet that a patient's safety depends.

Most operations are performed in the theatre, a large room with raised benches all round it, where the students may sit and see them; but those like the one that was now to be done—the removal of ovarian tumours and the like—are invariably performed in private, without one unnecessary eye to witness them.

Sister Grace was present, and another nurse, to whom the special charge of the case was to be given. The latter's work at present was to hand the operator the things he might require—needles, sponges, bandages, and the like. She had also to wash the sponges as they were used, for which purpose there was a lavatory arrangement in one window. Besides these, there was the operating surgeon, whose grave, strong, quiet face no one could fail to trust; a man who had given years of thought and experiment to the work he had now to do; and who had risked all manner of obloquy by determining to make the attempt to save the lives of women who were dying of tumour, while public opinion called out loudly that he was only killing them more quickly, and stigmatised him as rash and reckless. No one who looked at him, above all no one who saw the precautions he took before commencing an operation, could reasonably have thought him deserving of such a description; and now, when success after success crowned his efforts, praise came to him instead of blame—praise which left him as unmoved as the reverse had done. He knew too well the danger and anxiety belonging to every operation, to have any other feeling than humble thankfulness when all went well.

### Spray *v.* Germs: a Lilliputian Battle.

With him were the house-surgeon and two assistants, to administer chloroform, and manage the carbolic spray. This fine antiseptic vapour, falling continually during the operation upon all the parts exposed, prevents poisonous germs finding an entrance, and so lessens immeasurably the risk of suppuration and blood-poisoning. Few things were more mocked at on their introduction than this antiseptic treatment, which found enemies even among surgeons themselves, till its good results in hospital practice proved its value as a disinfectant. It ranks second only to chloroform in importance to surgery, for both have rendered practicable, with every hope of success, operations which otherwise it would be impossible to contemplate. It would be difficult to compute the lives that have been saved, the suffering that has been prevented, by chloroform and antiseptics; and he who introduced the antiseptic treatment deserves all the honour that can be bestowed on him.

The operation took more than half-an-hour. It was anxious work for all—for the surgeon, knowing that the faintest slip of the knife might be fatal; for the assistants, one with his finger on the patient's pulse to see that her strength did not fall too low, another keeping her under the full influence of chloroform, the third maintaining the continuous shower of spray; and for the nurses, standing ready to hand the operator whatever he required without a moment's delay. Such a task is not to be undertaken lightly; yet is it worth attempting when thereby a wife and mother, threatened with speedy death, may be spared to her dear ones for many years.

At last it was over. The patient was carefully transferred from the operating table to a water-bed; the doctor went away with a promise to return that evening to see how she was getting on; all traces of what had taken place were cleared away; and the nurse and house-surgeon remained to watch till the effects of the chloroform were gone. For two days Mrs. Johnson was more or less unconscious. She did not know that the doctor, after seeing his rich patients, came to the hospital and spent a whole night by her bed. She did not know how frequently the house-surgeon came in to feel her pulse, and mark the changes on her countenance. She did not know that she was never left alone for a moment, that by day and night a nurse trained to observe and attend to the slightest variations of a patient's condition watched over her. For many hours pain and weakness held supreme sway, and dulled all her faculties.

After a few days, however, she began to take note of her surroundings. The room was not large compared with the general wards, but it would have been thought of good size in a private house. It held two beds, a comfortable sofa, and a large table, yet did not seem crowded. Over the chimney-piece hung a good engraving, representing Christ blessing little children, and illuminated texts brightened the other walls. The engraving comforted Mrs. Johnson greatly, the subject appealed so strongly to her motherly heart. "Even if I should die,' she said, "God will take care of the children."

### An Old Acquaintance.

There was, however, no question of her dying: everything went well. On the Sunday following the operation she was able to see Aunt Ellen for a short time, though she could not talk much, and she soon began to feel quite at home in her new surroundings. To her surprise, the ward-maid, who scrubbed the floors and polished the grates, and washed up the breakfast and dinner things, proved to be an old acquaintance of hers. "Dear me, Lucy Jennings, is that you!" she exclaimed. "I thought you were married and had left service. Who would have thought of seeing you here?"

"I was married," answered Lucy, "and a sight of trouble I've had since, and been glad to get back to service again. But it's a long story, and I haven't the time to tell, nor you strength to hear it just now."

"I should like to hear it, though. Some day, when I'm a bit better, you may get an opportunity."

The opportunity came one day when Lucy had finished her work, and Mrs. Johnson was so far recovered that Sister Grace decided that to have a talk with an old friend would rather do her good than harm.

### A Sad Story.

"I said it was a long story," Lucy began, "but perhaps it isn't so much to tell as it was to go through. You knew, perhaps, that my George wasn't strong when I married him. I thought that perhaps it was just that he had been neglected a bit, living in lodgings, and not getting his food comfortable, and that if he had me to look after him he would be all right. I didn't know till we were married that his lungs were all wrong. His whole family had died of consumption, and he was going fast that way. He worked on as long as he could, but that wasn't very many weeks, and he was lying helpless for months. Well, well, it was a sad time, but the end of it was this, that he died not much more than a year after our marriage, and two months before my baby was born. What with the cost of George's illness, and no money coming in but the few shillings I could earn by sewing, we were very poor when the end came. There was rent due, more than the furniture we had left would pay, and my heart was like to break when I thought of the poor little child that would come into the world without a home or a father to provide one. Then the doctor advised me to go into the hospital till my baby was born, and promised to do what he could to help me afterwards. I didn't like the idea, I can tell you. I told him I didn't think it quite respectable; but he said that was all nonsense. 'You're not the only creature who has been poor and homeless at such a time,' he said. 'I'll get you into a hospital where you'll find plenty of married women who have as sad a tale to tell as you, and are as glad of a shelter as you can be.' And that was true. I can't tell you all the pitiful stories I heard in the hospital; but here's one. There was a poor girl there, quite young, and very pretty. She had been a governess, and had married a man who used to visit at her employer's house, and after she had taken him she found out that he had a wife already. She left him at once, ran away with hardly any money, and I fancy had a terrible time till some kind soul met with her, and brought her to the hospital. She said that she had just been saved from suicide, and I think it's likely enough. I did feel thankful that my baby would have the right to an honest name, and need never be ashamed of its dead father; and I could quite understand why she should be glad when some one offered to adopt hers. She cried terribly at parting with it, and she was always longing for it; and yet she was glad to let it go, all the more that it was a girl. That has nothing to do with myself, though. Well, my baby was born dead, poor lamb! and after I was well again the doctor got me the situation as house-maid in this ward; and very glad am I to have honest work to do, and to be able to do it."

"Well, Lucy, it's a sad story indeed. Somehow I never liked the name of lying-in hospitals; but may be there's something to be said for them."

"That there is, Mrs. Johnson. I have told you my own case, and there are plenty like it, when they're a blessing, and no mistake; and in other cases, where you can't respect the mothers, it is something that they are not tempted, as they might otherwise be, to kill their innocent children, just to get rid of them."

"He Prayeth Best who Loveth Best."

By this time Mrs. Johnson had become quite at home in the little ward. She no longer required the care of a special nurse; but she was never left alone for a long time. Sister Grace or one of the probationers was always coming in to see that she wanted nothing; and besides the daily visits of the house surgeon and the visiting doctor, she had the student-dresser who had charge of the case coming in to cheer her. He happened to be the same who had sung "Be still, my soul," in the large ward on the one Sunday she had spent there; and he always came into the private ward on Sunday afternoons and prayed with his patient, and sang a hymn to her and anyone who happened to be with her—a nurse, or perhaps Aunt Ellen and one of the little Johnsons. But though he was characterised by a depth of religious feeling by no means common in young men, he was not by any means grave. While he was bandaging his patient he would talk to her so pleasantly that she forgot to ask herself if the dressing of the wound hurt her, and he usually went away with some jest on his lips, simple enough in itself, and perhaps not very witty, but yet possessed of power to brighten some of the subsequent hours. It was he who, when she was getting strong, brought her the linnet that filled all the days with melody, and every week he gave her the *Graphic*, saying that she ought to like the perusal of it all the better for the aroma of tobacco smoke that lingered about its pages from his own reading of them. She was busy, too, for one of Sister Grace's visitors showed her, before she went away, how to make a knitted waistcoat that would be as good as an overcoat to Tom during the winter months; and she had persuaded Sister Grace to let her do a little mending of the ward linen—"just to keep her from forgetting how to hold a needle."

A Lady Patient and a Maid-of-all-Work.

"We are going to take you into the general ward again," said Sister Grace one day. "There is a new patient coming who will require this one to herself for awhile. She has just come home from Africa."

"From Africa?"

"Yes. Her husband is a missionary there. She fell ill and had to be sent home; and she says she will undergo any operation, run any risk, that will give her the chance of going back to him. She is a brave woman, is she not?"

"That she is! And I made such a fuss about coming here, and all my darlings only a few streets off, within my reach if I were dying and wanted to see them at the last!"

"Your fears were natural," answered the Sister. "But we have many cases here, both men and women, who will risk death for the chance of regaining the health on which the happiness, perhaps the bread, of those they love depends. These, however, are the cheerful cases. The saddest are those where the patients have had such a sad life that they long to die, and have to be cured in spite of themselves."

The general ward seemed very large and almost noisy to Mrs. Johnson after the private one, but it was bright and pleasant. Most of the faces in the beds were new, but those who had seen Mrs. Johnson before welcomed her as an old friend, with a kindly word or a smile. Only one girl, at the end of the ward, who was dying fast of consumption, did not even glance at her. Her mind was dull and wandering, but when occasionally it cleared, the chaplain, who was sitting by her bed, whispered words of pardon and peace, and hope for a future life. She was only a poor workhouse-bred maid-of-all-work, whose dreary life was thus ending in a lonely death; but in the hospital she had received all that could prolong her life and ease her sufferings, and now, when death was imminent, she had kind hands to smooth her pillow, and gentle voices to bid her look to a Divine Father's mercy and a Saviour's atoning grace. She died during the night, and before dawn her body was taken away through the long corridors to the mortuary. This was not the mere dull cell it is in many hospitals. The wife of one of the physicians had painted its walls in fresco, with figures of angels among palm branches and sun-rays, while interspersed were texts prophetic of resurrection. The place glowed with crimson, and blue, and gold, like an illuminated missal; a little altar stood at the end; on each bier lay a cross of gold-embroidered needle-work to rest on the dead bosom till the body was carried away to its last resting-place.

The Convalescent Home.

Mrs. Johnson looked around at the new faces, and asked for her old companions. "Where is Jessie?" she inquired.

"Jessie is gone to the Convalescent Home," replied Sister Grace. "You know there is a Home in the country to which we can send our patients when they are sufficiently recovered to leave the hospital, but not strong enough to begin work again. They remain there for three weeks, which is usually enough to set them up entirely. Unfortunately, the Home is not very large, and there are not funds either to increase its size or build a new one; so many patients have to go straight from the hospital to homes so poor or so imperfect in sanitary conditions, that before long they are sent back to us worse than they were at first, just because they had not accumulated sufficient strength to resist infection."

"And what happens when they don't get better, but just remain ill for months and years, as old people often do, and young people too, in some diseases that don't kill at once, and yet can't be cured?"

"We try to get them into the Home for Incurables; but that again is too small to take in all who need its benefits. Much is done for the sick, but how much more remains to do!"

"But about Jessie, Sister?" Mrs. Johnson went on. "Perhaps I am taking up your time with my questions; but I liked the girl, and it did seem a pity that she should go back to that rope-dancing business, especially when she hadn't strength to do it."

Sister Grace, who had been looking rather sad, smiled again. "I am happy to say," she answered, "that Jessie has got something else to do. She is to go into a biscuit factory. Her work will be simply packing the biscuits in tins, so it won't require much skill; and though the wages are not large they are sure. I had a letter from her father this morning, which you may like to read while I go to poor Mrs. Hodge, whose bronchitis is very bad to-day."

SIGNOR TOMMASSINO'S GRATITUDE.

While Sister Grace went away to arrange for a tent being put over Mrs. Hodge's bed, and the bronchitis kettle being set working to soothe her with its medicinal steam, our patient read the showman's letter. It ran thus—

"SISTER GRACE,

"Dear Madam,—My daughter has told us all you have done for her in the hospital, and how you are going to get easy work for her; and we are much obliged. Her mother and me has wanted to do something for you in return. We had heard of thanksgiving services, and we thought we would give a thanksgiving performance. And it has brought in seven shillings and fourpence, which is very good; and I have sent you the money by postal note. Business is pretty good just now, and I am,

"Yours respectfully,

"SIGNOR TOMMASINO."

"Well, now, if that isn't nice of him!" exclaimed Mrs. Johnson, when she had read the expression of the mountebank's gratitude. "If people like that can do as much, it shan't be said that Tom and me did nothing for the hospital in return for its curing me."

TOM CAN DO SOMETHING.

A way of showing some degree of thankfulness had already occurred to Tom's mind. He mentioned it to his wife next time he came to see her.

"I say, Mary, it has struck me," he said, "that though there are plenty of flowers inside the ward, there are none outside. I think I'll make a couple of window-boxes like what we have at home, and put in some crocus-bulbs and a slip of ivy at each side to train up the sides of the window. They'll all grow easily enough, and I do think a bit of green outside your window is a cheerful thing to look at."

"That's a good idea, Tom. Flowers are wonderful things to comfort one, as I've found often during the last six weeks. I think God must have been thinking specially of folks being ill and troubled when He made flowers. Often and often, when I've been worrying myself about you and the children, and how you would get on if I was taken away, I've looked at the plants in the ward, and said to myself, that if He took the trouble to create them and bring them into leaf and flower, he wouldn't forget you. But we must do more than that, Tom. We can't ever pay for the care I've had here, but we can do our best to secure it for other people. If we were to save a little—a very little, even threepence a week—out of your wages, it would make a fair subscription for the hospital at the end of the year, and we'd never feel the want of it, putting it away week by week. You'll do it, won't you, dear?"

"Gladly, Mary; though somehow I never thought of the like of us subscribing to a hospital. I fancied that was for rich people to do."

"Let them do their part, but let us do ours. I would like to help other poor women to be cured as I have been, and giving what we can spare to the hospital is the best way to do it."

"ISN'T THE HOSPITAL RICH?"

"But isn't it rich? Harry Dykes, who works with me, said that it was worth £15,000 a year. That'sa lot."

"It sounds a lot; but it can't be enough, for Sister Grace told me that there were two wards still unfurnished, because they hadn't money to keep them up. There's to be a concert for the patients held in one of them next week, and the doctor says I shall be well enough to go to it, and that I'll get home—just think of it, Tom!—the week after. But about the hospital funds. When I recall what I alone must have cost them, I see that they must need a lot of money—such beef-tea as I had! I never could have imagined anything so strengthening! and brandy continually when I was very weak; not to speak of the bandages and medicines, and there are 500 people always getting that."

She determined to ask Sister Grace about the matter.

"It is true we have some endowment," said the Sister, "and so are better off than the hospitals which depend entirely on voluntary subscriptions; but the assured income would not do much to cover the expenses of the place, which amount to £50,000 every year, in spite of the greatest care and economy in management. But the dispensary alone costs £5,000 a year. The greater part of that—£3,500—is spent on drugs; but there is £760 for spirit-of-wine, £460 for scientific appliances, £350 for ice, lemon-juice, and things of that sort. We use more than a ton of ice every day, so you see what a heavy item that becomes in the hospital expenditure. Then glass and earthenware alone cost £190 a year, a pretty large sum, you see. The £5,000 does not include the things required for surgical cases. £10 is spent on calico and flannel, £10 more on wool and tow, and £10 more on plaster. Surgical instruments, also, are a great expense. £500 a year goes on them, £250 on antiseptic appliances, £270 on lint, £100 on linseed-meal, and £50 on lard."

"Then the surgical cases cost a lot. How much will the hospital have spent on me?"

"Every case like yours costs at least £10. That is for the things necessary for the success of the operation alone, without counting the cost of food, and the like. Some of the beef-tea you had cost half-a-crown a pint."

"Good gracious! It's like swallowing money outright. Don't the patients feel surprised at their getting so much spent on them?"

"Some do," said Sister Grace smiling; "but they aren't all like you. Many of them seem to think the hospital exists by magic, and never even ask themselves where the money comes from to keep it up."

Mrs. Johnson felt guiltily that she had once been not much better than the patients the Sister spoke of; but she comforted herself with the thought that now she not merely cared to know where the money came from, but wanted to help in supplying it.

At last she was able to get up and walk; only a few steps at first, and these very tottering; but it was such a triumph to feel herself on her feet once more, that she declared that if she were a baby just learning to walk she could not be more proud of the accomplishment.

"And if I were your mother I could not be more delighted to see you do it," answered Sister Grace, ignoring the fact that, so far as years went, she might rather have been Mrs. Johnson's daughter. All the patients in her ward were her children, whether they were young or old—very troublesome children they were occasionally; but Sister Grace always made allowance for the pain they were suffering, and never lost temper with them.

THE STORE-ROOM AND THE MATRON.

It chanced that one day, towards the end of her stay in the hospital, Mrs. Johnson was sent by Sister

Grace to the store-room—the shop, as it was generally called—to fetch some tea. Such an errand was considered rather a privilege, as it proved that the patient had won the Sister's confidence; while Sister Grace found it a good way of giving the patients a little exercise preparatory to their going out.

The matron, a pleasant little lady of middle age, happened to be in the shop, and our patient, who, it will be seen, was somewhat garrulous, and did not hesitate to express her interest in all she saw, remarked what a large place it was, and how well and carefully everything was arranged.

"Order is the first law of a hospital, as of all other smoothly-going things," said the matron. "And it is large; but think how many people have to be supplied from it. We spend every year £6,000 on meat, £2,700 on milk, and £1,200 on bread. That implies a large family, does it not? Then there is £1,000 for eggs; another £1,000 for butter and cheese; £700 for vegetables; £550 for grocery; and £570 for fish. It takes all our endowment to keep up the kitchen alone."

"I should like to see your kitchen, ma'am," said Mrs. Johnson; "it would be a sight worth looking at."

"Come with me," answered the matron. "I am just going there, and you may see it."

### The Kitchen and the Cook.

The kitchen was a very large room, with its walls covered with white tiles, which showed every speck so plainly as to make perfect cleanliness a matter of self-respect. The cooking was done by gas and steam, which saved a great deal of cleaning up, as was proved by the fact that all the work of the kitchen was done by one cook, aided by a kitchen and scullery-maid. But then the method was perfect. Every afternoon the sisters sent their patients' diet-sheets to the steward's office, where the amount of each article required is calculated and ordered. Next morning the cook receives the lists of food required; and when the dinner is ready he assigns the gross quantity of each kind demanded for each ward. This is enclosed in hot-water boxes, which are taken off to a lift, that carries them up to the ward. In the little kitchen attached to each ward the probationers divide the food into the diets assigned to each patient, and serve it to them, thanks to the hot-water boxes and the speedy mode of transit, piping hot.

At that time the preparations for dinner were just commencing. The legs of mutton for that day's dinner were just about to be lowered into the hot safe where they were cooked, and the fish was ready for the baking or boiling it was to undergo. Eggs were being beaten up for the milk puddings, sago, rice, tapioca, and the like, that had been ordered, and potatoes were placed in nets which were to be hung up in a steam-chest to cook. Several probationers were assisting in the making of beef-tea; for some knowledge of cooking was part of the instruction the hospital gave its nurses, many of them having found, when they left the hospital and took up private nursing, that even highly-paid cooks did not always know how to make the dishes necessary for an invalid, and that it depended on themselves to see that their patients had suitable, dainty, and appetising food.

### An Everlasting Washing-day.

From the kitchen the matron led the way to the laundry, where there was a never-ending washing-day. Everything was done by machinery, or the work could not have been got through without the employment of a large number of hands; for every week about 6,000 sheets and 400 blankets were sent to the wash. After being steamed, and rinsed, and wrung, they were sent to the drying-ovens, and then passed under hot rollers by way of ironing. They were stored in a large room with wide shelves round it, and great open stands, through which a current of warm air passed continually; so there was little fear of damp sheets coming out of it.

"That is the coal-cellar at the end of the passage," said the matron. "We use about seven tons a day in winter. It takes the porters two hours to take them to the different wards; but you see we must have good fires. In another cellar there are splints for broken limbs of all sorts. We want a good many of those; we order them in by the hundred."

"Have you to attend to the getting in of all these things, ma'am?" asked Mrs. Johnson.

"Not to the splints and the like," answered the matron; "but I have to look after everything in the way of house-keeping—arranging for the meals of patients, nurses, and house-surgeons; taking care of the linen belonging to the hospital, besides choosing and managing servants, and so on."

"That's a great deal for any one person to do, is it not?"

"Happily, I can trust those around me," answered the matron. "I know that Sister Grace will see that everything goes right in her ward, and that the little kitchen at the end of it will be kept clean, and the work meant to be done in it not neglected in any way. That simplifies my task greatly. In a hospital, more perhaps than anywhere else, the conscientious work of all is necessary to success. Now take Sister Grace the tea she sent you for."

As Mrs. Johnson was going, a servant came up to the matron to say that the mattresses that had been cleaned had just arrived. The matron gave some directions about them, and turning, said to our patient, "We need to have all our mattresses cleansed and re-covered very frequently, for fear of any touch of poison lingering about them, which might infect a new patient. Now that we understand how infection is carried—by microscopic germs that may be hiding in the most unexpected places—we feel most strongly that cleanliness is the foundation of all cure. In fact, I think that if people could only learn to be thoroughly clean in their persons and their homes, many diseases would be totally unknown, which now swell the death-rate in an alarming way."

### The Concert and the Audience.

On that evening the concert took place, and though Mrs. Johnson was in a state of great excitement at the thought of going home next day, she was able to enjoy the music, and the strange, in some respects sad, spectacle presented by the audience. Some of those present were able to walk into the empty ward which was used as the concert-hall without any assistance, but the greater nnmber were compelled to hobble along, leaning heavily on a nurse's arm. One or two were brought in more stately style, rolling along in wheeled chairs; and several children were carried to their seats. Those present came mostly from the surgical wards, where the general health is not often affected by the accident or disease from which the patients are suffering. In medical cases there is usually too much danger to be feared from draughts, over-heating, and excitement to make it advisable for patients to be present at any of the entertainments. It was certainly the strangest assemblage that ever was "on pleasure bent." Legs stretched out in splints, or firmly imprisoned in plaster-of-paris bandages, alternated with heads gracefully turbaned with lint and carbolic gauze, decorated diagonally with bandages and oiled silk, that hid some damage to eye or cheek, or swathed in nun-like fashion, with the bandaging necessary to a broken jaw. There were several present who were suffering from cancer; all bearing on their faces the signs of the sad patience taught by that fearful disease, with the exception of one old cabman, suffering from cancer of the lip, brought on by much smoking of clay pipes, whose stems had been broken time after time, till little more than the bowl was left. He seemed rather at war with the fate that punished him so sharply for so trifling an indulgence.

There was no doubt that the concert was a great success. The house-surgeon, who had got it up, was himself a good violinist, and he had been at some pains to get together a number of musicians from among his friends. There was a good deal of part-singing, which gained special applause, perhaps because two of the nurses took part in it; and besides the pleasure which the sight of the familiar uniform seemed to give to all, those present who were under the singers' care felt an almost proprietary interest in the performance, which found vent in loud cries of "Encore," the word being pronounced in ways more or less original. Mrs. Johnson thought the piano solo a little dreary, not having been educated up to a comprehension of Beethoven; but the violin playing was delightful, and the singing more enjoyable still. Her pleasure rose to rapture when her own dresser—he who sang hymns in the ward—came forward and gave "Father O'Flynn," with a spirit that "brought down the house."

"Never did I think, when I entered the hospital," she exclaimed, "that I would spend such a happy evening in it before I left. I think the ladies and gentlemen who come here and make music for sick people must feel prouder than if their audience was the finest in the land. Don't you think so, Sister?"

"I think they are happier at least, because they have given pleasure to those who are suffering, and who perhaps have never had many amusements even when they were well."

So said Sister Grace, who had waited till nearly midnight, in order that she might see all her charges safely ensconced in bed after their unwonted dissipation, and be sure that none of them were injured by it.

### Little Tommy and his Hip-joints.

"By-the-bye, Mrs. Johnson," she added, "the house-surgeon was talking to-day of that little boy of yours—the lame one. He thinks it a pity he should be left in that condition. Won't you send him here, and let us cure him?"

"Oh, Sister! I can't! If anything happened to Tommy it would break my heart. I love him best of all the children, just because he is so delicate."

"I don't think there is any danger. It is simply a matter of taking out a piece of diseased bone, and thereby freeing him from a lifetime of gnawing pain. Just see how well you are. Are you not glad you came here, and won't you trust us to do as much for Tommy as we have for you?"

"There's no doubt about my being well. It's long since I have felt as strong and cheerful as I do now, and I really feel ten years younger. If he was to be under your care, Sister Grace, I shouldn't mind Tommy's coming; but I can't expect to find another nurse like you."

"When we are overcrowded with child-patients we sometimes put up cots in the general wards; but as a rule it is better to keep them in the children's ward. Sister Lucy, who has charge of it, adores children, and manages them in the most wonderful way. She says she is sure God meant her to be a mother, but gave her no children of her own, in order that she might have plenty of love to spare for other people's."

"If she is like what you say—but I don't know; I'll ask Tom about it," answered the mother, still dubious, afraid to let her child undergo one-tenth of the danger she had fearlessly risked herself.

Tom, however, received the idea gladly. There was a perfect festival when Mrs. Johnson returned home. All the children were in their Sunday clothes; Aunt Ellen, who had prepared a high tea of special excellence to welcome the mistress of the house, declared that, proud as she had been to sit at the head of the table and look after her brother and the children, she was prouder still to give up that seat of honour to one who had a better right to it than she. As for Tom, he said that his Mary looked younger and brighter, and prettier then than she did the day he married her; and when his wife disputed the veracity of the statement, he sealed it with a kiss.

So when she hesitatingly told him of the suggestion to take Tommy to the hospital and cure his lame leg, he said at once that it was the very thing to be done.

"But just think of the risk, Tom. There's always a little danger in these operations."

"Why, my dear, if I let you run the same risk, or a much bigger one, you don't think I would hesitate about the child. And just think what a blessed thing it would be for the poor little chap to grow up free from pain and able to walk straight. And I suppose if anything is to be done for his leg, the sooner it is done the better."

"Well, we are getting all we can out of the hospital."

"That's true; but we'll give all we can in return. If I have to go without 'baccy for the rest of my life to do it, I'll have a good sum to put into the offertory-bag on Hospital Sundays; and I daresay you'll try to save a bit in the housekeeping to add something to it."

Tommy cried a little when he was taken to the hospital, but by the time he was settled in his cot in the children's ward, he was as cheerful as possible.

"Oh! I say, mother, it's quite pretty here," he said, looking round the ward, where bright-coloured plates from the illustrated papers were interspersed with illuminated texts. "See, there's my little sweetheart, just like we have at home;" and he pointed to a copy of Millais' "Cherry Ripe," which hung on the opposite wall. "I shan't feel lonely with that to look at. And I'll tell you what to do, mother. You must always say good-night to the picture at home and think of me, and I'll say good-night to this one and think of you; and so we'll make believe that I'm not away from you at all."

### Sister Lucy.

"That's a very pretty idea," said Sister Lucy, who was standing by, "and I'll show you something that will help you to keep it in mind." She directed his attention to a glass globe near, which seemed to be filled only with water and water-weeds, but in which one could see several oval silvery-looking bodies that at the first glance looked like beads of quicksilver. Looking at them more closely, however, one could see that the beads vested and partly enclosed little animals, who rested peacefully, or scrambled in and out among the weeds.

"Oh! mother, look at the queer little creatures!" cried Tommy, almost shrieking with delight. "What are they, Sister Lucy? They're not fishes."

"They are water-spiders," explained Sister Lucy. "They live in the water, but not being fish, they can't breathe it, so they carry about with them this little bell of atmospheric air to live in. That's their home, you see, the place they could not do without (they get drowned if it is broken or taken from them), but they can carry it about with them. And your home, Tommy, is not so much the house where you live, as your love for your father and mother and sisters, and theirs for you; and that you can take with you anywhere. While you can always think of them lovingly and happily, and know that they are thinking so of you, you have your air-bell, your home, and need not feel lonely or miserable, though you don't see your parents and sisters every day."

Tommy caught at the idea at once. "I'll remember that," he said. "But will you let me always have the little spiders in sight, Sister, for fear I should forget, and feel lonely when mother's gone?"

"But I hope you won't feel lonely," answered the Sister. "You must make friends with those around you. Harry," she added, addressing the boy in the next cot, "this is Tommy Johnson; will you lend him your book or some of your toys?"

Harry looked scrutinisingly at Tommy. "He looks rather young," he said, gravely. "I don't know that he'd care for *Fighting the Flames;* but perhaps he would like a puzzle-map. Do you know geography?" he asked Tommy, condescendingly.

"Of course I do," was the rather indignant answer. "I've been at school for three years, and we're learning Asia just now, and I know the height of the Himalayas, and the population of Calcutta."

"Then I am sure you will like the map," interposed Sister Lucy, handing it over to him; and Mrs. Johnson left her boy quite happy, piecing together the bits of wood that formed the map of England.

The children made friends in their own fashion.

### "What is the Matter with You?"

"What's the matter with you?" asked Harry.

"I'm lame. They're going to make me walk straight, father says, and without my leg hurting me."

"Oh! then they'll have to give you sleeping-scent."

"What's that?"

"It has another name, but it's long, and I always forget it. It isn't bad stuff. It makes you sleep so sound that you don't feel anything, not though they were cutting your leg off; but you are a bit stupid and light-headed when you are waking up. I had it when they opened the gathering in my side—the 'absence,' the doctor called it."

"Is that what's the matter with you?'

"Oh! I've had lots of things the matter with me," answered Harry proudly. "It was the 'absence' when I came in first; and then I caught scarlet fever, and was taken to the fever hospital; and when I was getting better from that I took bronchitis, and that's just going away now. I've been here for months."

"Then you'll be going home soon?"

"N-no. And what's more, I'm jolly glad."

"Glad!" echoed Tommy in utter bewilderment.

"Yes. You see, I'm not like you. My mother's dead, and since I came here father has got into trouble"—this was the boy's way of alluding to the consequences of a burglary—"so I wouldn't have much of a home to go to. And as it chanced, I was put into Archie's cot, so I'm to have better luck than to be sent back to the old court again."

"What do you mean?"

### Archie's Cot.

"Do you see this?" Harry pointed to a brass plate, fixed into the wall at the head of his bed, on which the words "Archie's cot" and a date were engraved. "There's a lady who pays for the keeping up of this bed. Her own son died, and in memory of him she got her husband to give the money it would have cost to bring him up, to the hospital, to pay for a bed in the children's ward. She comes here every week and talks to all of us, but she always takes a particular interest in the one that's in Archie's cot. It was her that brought me that book—it's one of Ballantyne's, and it's first-rate, all about fires and firemen; it made me want to be a fireman myself. But I'm to be better than that, she says. She has got her husband to take me into his place, just as office-boy to begin with; but she says that if I'm industrious, and go to evening classes, I'll get on to be a clerk. Why, I don't know what I mayn't climb to be. I may get to be a big swell yet, and subscribe to the hospital myself! My, wouldn't I be proud! I would say that all the money I gave was to go to the children's ward; and I'd come often, and bring toys to the children, and play with them myself."

Meanwhile Mrs. Johnson had gone, begging Sister Lucy to be very kind to Tommy. "He has never had a harsh word said to him in all his life, being always delicate; and he couldn't stand it. He's so clever, too. I'm quite sorry to take him away from school, even for the time wanted for the cure of his leg; it will be such a vexation to him if he finds wh n he goes back that he has forgotten what he learned, and has fallen behind the other boys."

### School in the Ward.

"He need not do that. Having child-patients, who sometimes remain in the hospital for months, we try to keep up their education to some extent. Of course we can't set sick children to learn lessons, and we don't try to keep them up to the School Board standards. But we have a lady who comes twice a week and gets those of the children who are able for it to read to her; and then she explains the meaning of their books. She teaches some of them to write, too—in pencil only, for we must take care of the quilts. Then some of their toys are of a more or less instructive nature. We have several puzzle-maps, like that Tommy got, and boxes of letters with which the children amuse themselves by combining into words. What do you think of this, too?" asked Sister Lucy, taking up a book that lay on the cot of a sleeping child, and showing it to Mrs. Johnson. "It is written by the author of *Alice in Wonderland*, and gives arithmetical problems hidden in funny, fantastic stories, which amuse our little patients wonderfully. We don't let them become stupid here if we can help it; but you know we get many cases in which abstinence from study of any sort is the only cure. There is one—a girl of nine, laid up with brain-fever. She was getting on splendidly at school, we were told; was at the top of her class, though every other girl in it was older than she; and this is the result. Had she held out a few months longer the disease would probably have been fatal. She broke down just in time, and we shall be able to save her life. But very often clever children are encouraged to over-exert their brains, and permitted to neglect their general health to such an extent that either life or reason gives way. So don't grieve if Tommy does fall a little behind his classmates through his stay in hospital; it may be in every way the best thing that could befall him."

Handcuffs.

Mrs. Johnson was conscious that the warning was not undeserved; that she had incited Tommy to do his lessons even on the rare occasions when he had complained of headache or fatigue, though she knew well it was not laziness that made him idle. She turned the conversation by asking, "What has that poor little fellow done that he should have his hands tied? You surely don't punish the children, Sister?"

Sister Lucy laughed. "That isn't a punishment, but a precaution," she said. "He came in with a broken leg, and after it was set he would go on meddling with the splint and loosening the bandages. The result was that the bones did not join properly, and we had to break the leg again."

"Break it again!"

"Yes; otherwise it would have formed a false joint, and left him lame for life. So, to prevent his spoiling the mending of it this time, we tie his hands to the sides of the cot; not tightly, as you see, but with cords sufficiently short to prevent his touching his leg."

A farewell glance at Tommy, and the assurance that she knew he was in good hands, and then Mrs. Johnson went away, having received permission to return on Sunday.

"No, Sister Lucy isn't just the same as Sister Grace; but she is just as nice," she said to Tom when she got home. "She is more merry; seems to be inclined to laugh at everything, and seems as fond of the children as if they were all her own. Tommy is in good hands."

Tommy fixed up.

Still she was a little puzzled at the attitude in which she found Tommy when she paid her next visit. The "sleeping-scent" had been administered by that time, and the decayed bone taken out of the leg. His head was well propped up on pillows; but the lower part of the bed was raised some inches above the head of it, so that the child lay in a sloping position with his feet above his body.

"That doesn't look very comfortable," the mother observed.

"It is necessary," explained Sister Lucy. "There is always some risk of the leg contracting when a bone is excised; so, to prevent that, we fix a weight to the foot by a string. Here it is, hanging at the foot of the bed; and in order that it should bear as much as possible on the affected part, we have to raise the bed in this fashion."

"I'm quite comfortable, mother," added Tommy. "I have *The Boy Crusaders* to read, and Harry gave me his draughts before he went away, and showed me how to play. He says he is going to be a business man now, and won't want to play at any games."

"Archie's cot" had a new occupant now, a crowing, merry baby of about a year old—merry, in spite of a famished look which had not yet disappeared from its little face.

"A deserted child," said Sister Lucy, by way of explanation. "When it was brought here two days ago I thought it was dying. I could hardly feel its heart beat, and it had bronchitis very badly. But we steamed it with the bronchitis-kettle, and gave it plenty of new milk, and now I think it will live after all." As she finished her explanation, the Sister bent down to talk sweet, incoherent baby-language to the child, who laughed in response and stretched out his hands to the Sister, as if he had known and loved her all his little life.

A Diminutive Colonist.

"But what will you do with him after he is well?' asked Mrs. Johnson.

"Send him to Canada, probably. Emigration is the best thing for these homeless children."

"But you can't emigrate a baby like that."

"Not yet; but it is better to send them out while they are quite young; they make better colonists when they have been brought up in the country in which they are to make their home."

"Well, it seems hard to send mere infants to a foreign land."

"I wish we could do it oftener," said Sister Lucy.

"I'll Break my Leg."

"Anything would be better than the homes many of the children have to return to. Just imagine, you who are a mother, all that it means when a child cries at the idea of returning home, and begs to be allowed to remain in the hospital. They make the most of their ailments, many of them, sly little creatures that they are,—cough and sob and try to look as ill as they can when the doctor pays his visit; but one can't feel very angry with them. One little fellow amused me greatly, though I had to look severe at his speech. When he went out a few days ago he asked me to keep his cot for him, as he meant to break his leg as soon as he could, in order to get back."

"And will he do it?"

"I don't know; but I suspect he may; for he has been here three times already, at very short intervals, having always met with some accident. He is a rickety child, anyhow, with too much lime in his bones, the doctors say; but I suspect him of taking no special care to avoid damaging himself."

"Then will you keep his cot for him?"

"Of course not. It is against the rules, and moreover, it isn't practicable. There are always claimants for every vacant bed. His has a new tenant already. Just look at him; is he not a sad spectacle?"

A Boy Tramp.

She directed Mrs. Johnson's attention to a boy of about ten, who was sitting up in his cot, listlessly turning over the pages of a Scripture picture-book.

"He is a tramp's child," said the Sister. "He had never lain in a bed till he came here, and could not recollect having been washed. The result is that he is afflicted with a skin disease, which it will take a long time to cure."

"Good gracious!" Mrs. Johnson shrank away little from the cot, and felt a little uncomfortable at Tommy being within twenty yards of the vagrant's child. "And who do you get to wash him, Sister? It can't be a pleasant task."

"It isn't, so I do it myself. I don't like to set a probationer to a task like that; they can't always help showing their repugnance to it. Moreover, he is very tender, and requires careful touching."

"Well; he ought to be grateful to you, that's all. Is he?"

"Not yet; we shall come to that; but at present he is too unused to receiving any kindness to realise that it is given without some evil design. He distrusts everybody; but I have no doubt he will be quite humanised before he leaves us. You have no idea how ignorant he is. He had never heard of God; and, of course, had no idea that it was wrong to lie or steal. The chaplain has been talking to him; but it is difficult to make way against such uncomprehending ignorance as his. I think he will learn better after he has been here a week or two. When he knows that human beings can be kind to him, he will be better able to realise the love of God."

Little Nellie.

While they were speaking, a little girl, a pretty, golden-haired child of six, came up to Sister Lucy. "Please, Sister, may I go and sit by poor Jack? I want to show him my book. It's about Daniel, and I think he would like to hear about the lions."

"Certainly, Nellie," answered the Sister, and she brought a tall chair from the end of the ward, and installed Nellie in it by the side of the little tramp's bed. The boy smiled at her, although, from sheer lack of experience in courtesy, he had no other word to offer her than a curt—"What do you want?" In reply Nellie opened her picture-book and began to explain the meaning of its highly-coloured plates to the best of her ability.

"The humanising influence has begun," said Sister Lucy. "I don't know why Nellie has taken to the poor fellow so much; but there is no doubt that she is doing him a great deal of good. Unfortunately, she is going out in a few days."

The Cost of Tracheotomy.

"You'll be sorry to lose such a sweet child," remarked Mrs. Johnson. "You must be fond of her."

"Yes, we all are; though she has cost us more than we care to think of."

"How, Sister?"

"She was a tracheotomy case; that is, when she came in she was suffering from a disease which caused her throat to be covered with a thick layer of mucus, so that she could not breathe in the ordinary way. The only remedy was to make an opening in the throat and insert a tube by which to convey air to the lungs; otherwise she would have died of suffocation. Unfortunately, when the tube was put in the mucus filling up the throat was so thick that the air could not penetrate, so our house-surgeon sucked out the stuff—it was rank poison—through the tube, and cleared the orifice. He saved her life, but at the cost of his own. He took the disease and died in a few days."

"You don't mean to say that he did that, knowing it would kill him."

"The thing has been done without evil result; but he knew the danger of it; he knew he was risking his life in what he did."

"Why, it was martyrdom!" cried Mrs. Johnson.

"It was only a not uncommon example of hospital heroism," answered Sister Lucy.

A Street Accident.

The next time Mrs. Johnson went to see Tommy there was patient of a new sort in the ward, not far from him—a tiny boy, not more than three years old, who had just been brought in, and was crying pitifully for "Mamma" and "Nurse."

Sister Lucy was trying to comfort him, and the house-surgeon was standing by his cot. A probationer explained to Mrs. Johnson what had happened.

"A street accident. We can't gather from him exactly how it happened; but it seems that he lives in another part of the town from this, and that his nurse brought him a journey by train, and said she was going to meet a friend. The friend met them at the station, and while the two were talking the child slipped away, and somehow fell in front of a cab and was run over. His leg is broken; but the shock to his nerves is really the more serious injury; it seems quite impossible to soothe him. We have found out that his name is Freddy Charteris; but he can't tell us where he lives, except that it is a long way off, and near a park. We must trust to the nurse going back and telling of her lost charge. She won't do that till she has made search for him herself, and as this may not be the first place where his parents will look for him, he will perhaps be here all night. Poor little fellow! I hope he won't cry himself ill."

"I Knowed You'd Come for Me."

But even while the probationer was speaking a rustle was heard outside, and a lady was ushered in by the matron, who asked—"Have you a patient called Freddy Charteris here, Sister?"

But before Sister Lucy could answer the newcomer had caught sight of Freddy, who exclaimed, "Mamma, I knowed you would come for me!" at the top of his tiny voice, and threw his little arms round her neck. For a few minutes she could do nothing but clasp him to her breast and kiss him, while he explained how he had lost "nussie," and the horse knocked him down, and a man picked him up and brought him here, where they had tied up his leg in pieces of wood, and it hurt him very much. When he had become calmer, however, and was sobbing more quietly, she turned to the matron and Sister Lucy.

"You will think me very discourteous," she said; "but I was so thankful to see my child again, that at first I observed no one else. You may imagine how terrified I was when the maid came home and told me she had lost him; I never thought to see him alive again. My husband, Sir John Charteris, has gone to the police-office to inquire about him; but happily my doctor, who was in the house when the news came of Freddy's being lost, advised me to come here. I can't be too grateful to you for taking care of him."

When Lady Charteris mentioned her husband's name Mrs. Johnson started, and said, scrutinising her features closely, "Can it be really our Miss Eleanor? Yes, I do think it is! I know it was one Sir John Charteris she married, but I haven't seen her since she was only a little girl herself."

"How, Mrs. Johnson, do you know this lady?" asked the house-surgeon, who was standing near her, having withdrawn from his position by Freddy's cot when Lady Charteris appeared.

"If she was once Miss Eleanor Nugent, I do; and I was her nurse till I married, and I would have been torn by wild horses before I would have let her come to any such accident as befell her poor little boy to-day."

Lady Charteris caught the sound of her old name, and turned towards the group. For a moment she scanned Mrs. Johnson doubtfully, but she soon recognised her. "Why, nurse, is it really you! How strange that I should meet you here again. Are you connected with the hospital?"

"Oh, no, Miss—I mean, my lady—I just came in to see my little boy, who is here being cured of his lameness."

"Oh, yes! I remember now. You married that nice carpenter who came to mend the nursery furniture when we children had knocked it about a good deal. I wish you hadn't. I remember how careful you used to be not to let us out of your sight; so unlike that girl through whose carelessness my poor little Freddy so nearly lost his life. I wish I could get you or some one like you to take care of him. Do you know any reliable person I could get to take her place?"

"There's my sister-in-law, Ellen, my lady, if one may speak in praise of their own. She's as nice a girl as you'll meet anywhere, and thoroughly trustworthy, as I know; for she left a good situation to take care of my husband and children when I was ill, and in the hospital here for two months, and they were as well looked after by her as they could have been by myself."

"Oh! do send her to me," said Lady Charteris. "I will engage her on your recommendation; I am sure that she will be all that I want."

"Now, don't things work round strangely!" said Mrs. Johnson to herself, when she had thanked Lady Charteris, and promised that Ellen should call at Marchmont Square next day to be interviewed by her future mistress. "Who would have guessed that when Ellen gave up her situation to oblige me it would end in her getting a much better one? for unless Miss Eleanor is very much changed she'll be the kindest mistress in the world."

Freddy, meanwhile, had sobbed himself to sleep, and Sister Lucy asked Lady Charteris to come into her sitting-room and have some tea, while the house-surgeon sent a telegram to Sir John to let him know that his son had been found, and ordered the ambulance waggon to be got ready to take the little patient home.

"What a charming little room!" exclaimed Lady Charteris, as she entered Sister Lucy's sanctum. And indeed it was very pretty, though, as she soon noticed, there was nothing at all costly in its furnishing. But there were flowers and ferns on the window-sill, and one or two pretty pieces of china on the chimney-piece, and the simple decorations were so exquisitely dainty and neat, that it could compare favourably with much more finely-furnished apartments.

### A Grateful Baronet.

The losing of Freddy Charteris proved to be a great blessing to the children's ward of the hospital. Sir John Charteris sent down a splendid hobby-horse, and Lady Charteris a beautiful case filled with lovely ferns; and they endowed a cot in the ward in memory of the kindness shown to their little son within its walls.

"But that must not be the end of what we do to show our gratitude," said Sir John, when he and his wife were talking over the matter. "I went over the hospital the other day with the house-governor, and I was utterly surprised to see what a vast place it was, and how admirably it was managed. The good it does is incalculable, and yet more remains undone for lack of means. 'If we were to do all we should like,' said the governor, 'we should spend our income five times over. Occasionally you will see in the newspapers a sensational paragraph about a case being taken to the hospital and refused admittance. You may be sure that such a thing occurs only when the hospital has not a bed to spare nor money to provide one. We have here two wards unfurnished, because our income does not more than cover the expenditure on those that are in working order. Hospitals are like individuals, and must be chary of running into debt from even the best of motives.'

### Hospital Sunday: a Drop in the Bucket.

"'But I suppose the Hospital Sunday Fund helps you a good deal,' I said.

"'We are glad of what we get from it,' he answered; 'but when you reflect that all that London subscribes on Hospital Sunday would barely suffice to keep this one hospital for a year, you will see that, however much is done, more remains to do. People don't seem to see that what they give to the hospital may perhaps be saved from the workhouse; that it is better to restore a working man to health than to let him die and keep his widow and children as paupers; or that it is wise to save the life of a mother whose children, deprived of her care, may develop into vagrants or criminals; and then there's the training of nurses and doctors. Their own lives may one day depend on the efficiency of the training the hospital gives, and yet won't give anything to maintain that efficiency.'

"I felt rather guilty, as you may imagine, for I don't think I have ever given as much as a guinea a year to a hospital in my life, so I said that perhaps people didn't know how much good they did.

### "They Don't Know."

"'That's it,' he answered; 'they don't know, or at least don't realise, the value of hospitals. Occasionally one or another of them gets put before the public in a special manner, and profits by it. If it has influential friends, they get up a fashionable bazaar in its aid, or have one of the Royal Family to open a new ward, or something of the sort. That's good, and we are grateful for help of any sort; but it seems a shame that people require to be so wheedled out of their money for such an object. Certain institutions gain more sympathy than others—hospitals for women and children, for example; and that is well, seeing that these are helpless creatures on whom more than their fair share of the suffering of humanity seems to fall—but all need help. Those who know the work done give largely according to their means. You would be surprised at the efforts, the sacrifices, made by some of the poor to repay the hospital for any benefit they get from it'—and he instanced the old nurse, Eleanor, and her husband, who seemed to be grateful people—'and when rich people become acquainted with the working of the institutions, they do their part, as a rule. But there is a large class of people who are always pretending to be better and richer than they are—the people who "keep up appearances,"—and these have never anything to spare for charity.' So I thought, my dear, that as we at least don't belong to that class, we might very well do something for the place where our child was so kindly treated; and I offered to furnish and endow one of those empty wards."

A Privilege—Not a Task.

"I am so glad you did," answered Lady Charteris. "Mrs. Johnson told me all that they did for her in the hospital, and it does indeed seem wonderful, almost miraculous, that such cures as that of her case could be possible. She spoke so quaintly of it all, and with such real great feeling, that I was greatly touched. 'It was my case last, my lady,' she said; 'it may be yours next; and if it were, it would be out of a hospital that the knowledge and skill to cure you would come. What you give to the hospital you are lending to the Lord, and may receive again some day with interest; though for my own part,' she added, 'I'll have to be in debt to it to the end of my days. All that I can do and give will be too little to repay what I and my child have got from it.' What she says, John, is quite true, and I should like if you would arrange for this ward you are going to endow being one for women. And for Freddy's sake we won't forget the children's ward either. Women and children, dear, are those whom men are bound to protect. The day of fighting for them is past, but in trying to help and restore them when they are laid down with disease, every man may do knightly and noble service. The doctors do their part, and do it well; other men can but play the squire's part, and equip them for their service; but that, I think, is worth doing; it is not a task, but a privilege."

So spoke Lady Charteris, and her husband agreed with her. Is not her point of view one which every woman may share who knows the special dangers that belong to her sex? May not her words find an echo in the heart of every man who has, or hopes to have, wife or child of his own, for whose sake he will do what he can for other women and children? Is any other incitement needed? Then let it be remembered that they who help the sick are specially mentioned among those to whom it shall be said: "Come, ye blessed of my Father; inherit the kingdom prepared for you."

